# Supplementary material for: Brainstem evoked auditory potentials in tinnitus: A best-evidence synthesis and meta-analysis
Source: Front Neurol. 2022 Aug 22;13:941876. doi: 10.3389/fneur.2022.941876 (PMC9441610; doi:10.3389/fneur.2022.941876)
Supplement: Supplementary file 1 [file Data_Sheet_1.docx]

Supplementary Material

# S1: Search strategies for PubMed and Web of Science

**Search strategy for PubMed:**

((tinnitus [MeSH] OR tinnitus [tiab] OR phantom sound* [tiab] OR ringing [tiab] OR buzzing [tiab]) AND (Evoked Potentials, Auditory [MeSH] OR auditory evoked potential* [tiab] OR event related potential* [tiab] OR frequency following response* [tiab] OR Auditory Evoked Response* [tiab] OR Cochlear Microphonic Potential* [tiab] OR Auditory Brain Stem Response* [tiab] OR Auditory Brain Stem Evoked Response* [tiab] OR Acoustic Evoked Brainstem Potential* [tiab] OR Auditory Brainstem Evoked Response* [tiab] OR Acoustic Evoked Brain Stem Potential* [tiab] OR Auditory Brainstem Response* [tiab] OR Short Latency Response* [tiab] OR Brainstem Evoked Response Audiometr* [tiab] OR Brain Stem Evoked Response Audiometr* [tiab] OR Brainstem Auditory Evoked Potential* [tiab] OR Brain Stem Auditory Evoked Potential* [tiab] OR Middle Latency Response* [tiab])) NOT ("review"[Publication Type] OR "systematic review"[Publication Type] OR "meta analysis"[Publication Type])

**Search strategy for Web of Science:**

TS= ((tinnitus OR "phantom sound*" OR ringing OR buzzing) AND ((auditory OR acoustic) NEAR/3 (evoked OR "brain stem" OR brainstem) NEAR/3 (potential* OR response*) OR (short OR middle) NEAR/3 (latency) NEAR/3 (potential OR response) OR "event related potential*" OR "frequency following response*" OR "Cochlear Microphonic Potential*"))

# S2: Data extraction tables

## Normal hearing (PTA ≤ 20 dB HL)

| **Reference (first author, journal citation, year)** | **Subjects** | **Control** | **Exclusion criteria** | **Outcome measure** | **Results** | **Comments** |
| --- | --- | --- | --- | --- | --- | --- |
| ABR | | | | | | |
| Barnea et al., Audiology, 1990 (82) | TI (n=17)  - ♂/♀ not given  - MA = 35 y  - CNH  - Mild to moderate TI  - Mean TL at TP frequency: 15.3 dB SL | No TI  - ♂/♀ not given  - Age not given  Age, sex and audiometrically matched | Unclear | ABR (lat and amp of waves I, III, V and IPL V-I)  1024 alternating polarity clicks of 100 µs, 120 dB SPL (and subsequently at threshold intensities in 5 dB increments), 10 clicks/s, presented binaurally  Bandpass filter: 200-2000 Hz | No sign differences in lat and amp of waves I, III, V and IPL V-I at 120 dB SPL | 12 out of 17 TI subjects participated in ABR evaluation |
| Cartocci et al., International Tinnitus Journal, 2012 (89) | TI (n=10)  - all ♂  - MA = 43.9 ± 11.04 y  - 5 BL TI, 5 UL TI  - CNH | No TI (n=14)  - all ♂  - MA = 45.1 ± 11.95 y  - CNH  Age matched | - Psychiatric pathologies  - Neuropathy  - Substances of abuse and serotonergic drugs assumptions  - Other major pathologies | - ABR (lat waves I, III, V and IPL I-III, III-V and I-V)  Series of 90 and 80 dB HL stimuli (clicks), stimulus duration 100 µs, 11 clicks/s, epoch duration 10 ms  Bandpass filter: 150-1500 Hz | Sign longer lat peak V and interpeak III-V lat with intensity of stimulation of 80 dB in TI vs control (peak V lat: TI 5.932 ± 0.191, control 5.746 ± 0.213; interpeak III-V lat: TI 1.979 ± 0.109, control 1.837 ± 0.198) | ABR latencies correlated with sleep and anxiety in TI |
| Dadoo et al., International Tinnitus Journal, 2019 (84) | 80 CNH ears, TI  - 46 ♂, 34 ♀  - MA = 33.41 ± 11.27 y  - BL TI in 28 subjects, UL TI in 24 subjects  - CNH  - Normal otoscopic findings  - Mean TI duration: 6.47 ± 8.37  months | 80 CNH ears, no TI  - 46 ♂, 34 ♀  - MA = 30.64 ± 10.39 y  - Normal otoscopic findings  No info on matching | - Hearing impairment, PTA > 25 dB  - Age ≥ 55 y  - Any external or middle ear disease  - History of intake of ototoxic drugs or anti-tubercular drugs  - History of associated psychiatric illness  - History of noise exposure or acoustic trauma  - Any chronic medical illness | ABR (lat of waves I, III and V and IPLs I-III, III-V and I-V)  Click stimulus, 10-30 clicks/s, 80 dB  Each ear assessed individually | - Latency of wave I sign prolonged in TI ears  - Latencies of wave III and V prolonged, but not sign in TI ears | - Individual ear as study unit  - Short duration of TI complaints |
| Dos Santos-Filha et al., Clinics, 2014 (90) | TI (n = 30)  - 26 ♂ and 4 ♀  - MA = 41 y  - CNH  - Exposed to occupational noise (> 85 dBA) | No TI (n = 30)  - 26 ♂ and 4 ♀  - MA = 41.6 y  - CNH  - Exposed to occupational noise (> 85 dBA)  Age matched | Neurological, psychiatric and behavioural dysfunctions | ABR (lat of waves I, III and V and IPLs I-III, III-V and I-V)  Click stimulus with rarefied polarity, 80 dBHL, 19 clicks/s, 0.1 ms duration, 2000 stimuli, 2 measurements on each side | - TI: No sign diff for R an L ears  - TI vs control:   - No sign diff in absolute latencies - Sign diff in the normal and altered responses   More ABR-alterations in the lower brainstem |  |
| Gabr et al., Audiology & Neurotology, 2020 (91) | TI (n=30), CNH  - ♂/♀ not given  - Age = 18-40 y  - TI duration: 5.8 ± 2.3 y  - THI 35.2 ± 16.9 | No TI (n=30), CNH  - ♂/♀ not given  - Age = 18-40y  Age and sex matched | - Hearing threshold level > 20 dB HL in the above frequency range  - Middle ear disorders  - Suspected retrocochlear pathology  - History of ototoxic drugs or noise exposure  - Neurological or psychiatric diseases  - Not in age range 18-40 yrs | ABR (amp and lat waves I, III, V)  Click stimuli, 90 dB HL, 21.1/s, 1024 sweeps. Monaural and binaural recording | Monaural:  - sign ↑ lat waves I, III and V in TI  - sign ↓ amp wave I and III in TI  - Later waves: trend toward ↑ lat and ↓ amp for later waves as well (though not yet sign)  Binaural:  - sign ↑ binaural wave I and III lat in TI  - sign ↓ wave I amp in TI |  |
| Hsu et al., B-ENT, 2013 (92) | TI, CNH (n = 15)  - 7 ♂, 8 ♀  - MA = 41.1 ± 12.9 y  - UL TI (n=7), BL TI (n=8) = 23 TI ears  - Persistent tinnitus of unknown etiology | No TI, CNH (n = 15)  - 5 ♂ 10 ♀  - MA = 37.9 ± 10.9 y | - Systemic diseases  - History head injury  - Neurological or otological complaints  - Long-term history of congenital HL  - Psychological comorbidities | ABR (lat wave I, III, V and IPLs)  Click stimuli, 80 dBnHL, rate 11.1/s, average of 1024 sweeps. | - Sign longer latencies waves III and V in TI  - No sign diff in IPLs |  |
| Kehrle et al., Archives of Otolaryngology - Head and Neck Surgery, 2008 (67) | TI (n = 37)  - ♂/♀ not given  - MA = 36 ± 7.2 y  - CNH, normal impedance audiometry (type A)  - Moderate to severe and disabling tinnitus  - UL TI (n = 13), BL TI (n = 24) = 61 TI ears | No TI (n = 38)  - ♂/♀ not given  - = 76 ears  - CNH  Age and sex matched | - middle ear, cardiovascular, or neurologic diseases  - Noise exposure or acoustic trauma  - Medication use | ABR  - lat of waves I, III, and V  - interpeak latencies I-III, I-V and III-V  - V/I amp ratio  - Interaural relation of lat of wave V (2 subgroups: UL TI and BI TI)  100 µs click stimuli, 1000-2000 alternating polarity clicks, 2-4 kHz, 80 dB nHL, 12 clicks/s. Contralateral masking 50 dB nHL. Filter: 100-2500 Hz | - sign ↑ lat of waves I, III and V in TI group  - interpeak III-V sign ↑ in TI  - V/I amp ratio sign ↑ in TI | No. of ears considered instead of no. of patients |
| Konadath et al., Intractable & Rare Diseases Research, 2016 (85) | TI (n = 20)  - 10 ♂, 10 ♀  - MA = 33.15 ± 9.80 y  - CNH  - Score > 38 on THI | No TI (n = 20)  - 10 ♂, 10 ♀  - MA = 20.50 ± 1.79 y  - CNH  No info on matching | - History of middle ear infections, use of ototoxic drugs or significant noise exposure | ABR (amp and lat of waves I, III, and V)  100 µs monoaural click stimuli, 11.1 clicks/s, 70dB nHL, filter 30-3000 Hz, 1500 sweeps | No sign diff in lat and amp |  |
| Makar et al., International Tinnitus Journal, 2017 (68) | TI (n = 30)  - all ♂  - MA = 39.61 ± 3.56 y  - age < 45 y  - CNH  - UL TI ≥ 6 months  - Mean TI duration: 15.06 months ± 8.76  - TP: n = 19 < 4 kHz, n = 11: 4-8 kHz  - Normal middle ear function | No TI (n = 30)  - all ♂  - MA = 37.59 ± 4.14 y  - age < 45 y  - CNH | History of any otological, psychological or neurological problems | ABR  - Lat of wave I, III and V  - IPLs I-III, III-V and I-V  Alternate polarity click stimuli, 95 dB nHL, 19.3 clicks/s, 2000 sweeps, bandpass filter 150-3000 Hz | - lat wave I, III and V sign ↑ in TI  - IPL wave III-V sign ↑ in TI (I-III and I-V no sign diff) |  |
| Nemati et al., Acta Medica Iranica, 2014 (83) | TI (n = 25)  1) TI UL, CNH (n = 19)  2) TI BL, CNH (n = 6)  - 9 ♂, 16 ♀  - MA = 34.4 ± 12.2 y | No TI (n = 16)  - ♂/♀ not given  - MA = not given  Matched for age, gender and hearing thresholds | - Pulsatile TI  - Hearing thresholds > 25 dB HL  - abnormal (no type A) tympanograms  - History of exposure to hazardous levels of noise  - History of ear surgery  - Ototoxic medications | ABR (amp and lat waves I, III, V; IPL I-III, III-V, and I-V; amp ratio III/I and V/I)  Alternating polarity clicks, 2000 sweeps, 90 dB SPL, rate 11.1 clicks/s | - No sign diff in lat of waves I, III and V  - No sign diff in interpeak lat of I-III, III-V and I-V  - No sign diff in amp of waves I, III and V and in III/I amp ratio  - Amp ratio III/I: No sign diff  - Significantly ↑ V/I ratio in TI |  |
| Omidvar et al., Otology & Neurology, 2018 (60) | TI (n = 18)  - 9 ♂, 9 ♀  - MA = 38.11 ± 8.80 y  - CNH  - Continuous TI for ≥ 6 months in right ear  - THI: n = 9 slight TI severity, n = 9 mild TI severity  - TP and TL | No TI (n = 20)  - 9 ♂, 11 ♀  - MA = 35.55 ± 8.29 y  - CNH  Matched on age, sex and hearing sensitivity | - Abnormal hearing sensitivity (≥ 25 dB HL)  - Abnormal results of acoustic reflexes and middle ear tympanometry  - Precedent of audiological, psychological, neurological, or medical complications | ABR (lat and amp waves I, III, V, and Vn; IPL I-III, III-V, I-V, V-Vn)  Alternating polarity clicks, 100 µs, 80 dB SPL, rate 13.3 clicks/s, 2 blocks of 2000 artefact-free sweeps  sABR (FFR) (onset (V and A), consonant-to-vowel transition (C), offset (O), and FFR (D, E, and F) peaks; amp F0, F1, HF)  40 ms synthesized syllable /da/, stimulus started with 10 ms noise burst with central frequencies close to the initial frequencies of the third to fifth formants in the 2580-4500 Hz range. Rate 10.9/s, 80 dB SPL. Bandpass filter: 100-2000 Hz. 1024 digital sampling points over 85.33 ms epoch. 2 blocks of 3000 artifact-free sweeps recorded in right ear. | ABR  - mean lat of waves III, V, and Vn sign ↑ in TI  - IPL I-V sign ↑ in TI  - No sign diff in amp of waves  sABR (FFR)  - Mean lat of waves V, A, C, D, E, F, and O sign longer in TI  - Mean slope of V-A complex and mean amps of F1 and HF  - No sign diff in mean duration and amp of V-A complex, amp of F0. | - Blinded investigators |
| Schaette et al., The Journal of Neuroscience, 2011 (73) | TI, CNH (n=15)  - all ♀  - MA = 36.3 ± 2.6 y  - > 6 months stable TI  - TI loudness and pitch matching was done, rated their TI on scale from 0-10 | No TI, CNH (n = 18)  - all ♀  - MA = 33.2 ± 1.9 y  Age and gender matched | - TI < 6 months  - Pulsatile TI | ABR  - amp of waves I and V  50 µs clicks at 90 and 100 dB SPL, 11 clicks/s.  Bandpass filter: 100-1500 Hz.  Averaged: ≥ 8000 repetitions for 90 dB SPL, ≥ 6000 repetitions for 100 dB SPL | - sign ↓ amp of ABR wave I in TI  - no sign diff in amp of wave V |  |
| Shim et al., PLoS One, 2017 (93) | UL TI, CNH (n = 43)  - 19 ♂ (MA= 28.58 ± 10.88 y)  - 24 ♀ (MA = 37.58 ± 14.38 y)  - Mean TI duration ♂: 7.61 ± 16.66 months  - Mean TI duration ♀: 7.15 ± 13.25 months | No TI, CNH (n = 18)  - 8 ♂ (MA= 28.50 ± 6.97 y)  - 10 ♀ (MA = 28.70 ± 11.78 y)    Age and gender matched | - Suspected of having objective tinnitus or somatic TI  - Chronic otitis media, retrocochlear lesions, endolymphatic hydrops, or congenital ear malfunction  - TI accompanied by dizziness  - History of ototoxic drug use | ABR  - amp and lat of wave I and V  - wave V/I amp ratio  - wave I-V interval  Click stimuli, 90 dB nHL, 13.3 clicks/s, 1500 sweeps. Average data from 2 trials | - ♂: sign ↓ amp of wave V in TI ears vs controls  - ♀: no sign diff |  |
| Shim et al., Otology & Neurology, 2021 (94) | UL TI, CNH (n = 27)  - 12 ♂ (MA = 32.83 ± 12.63 y)  - 15 ♀ (MA = 39.8 ± 11.2 y)  - Mean TI duration: 183 ± 322.5 days  - Mean pitch: 4.8 ± 3.4 kHz  - Mean loudness: 8.3 ± 4.4 dB SL  - Mean THI score: 41.6 ± 26.0  - Mean VAS of TI loudness: 5.7 ± 2.1  - Mean daily TI awareness 60.4 ± 32.8 % | No TI, CNH (n = 27)  - 12 ♂ (MA= 32.2 ± 5.3 y)  - 15 ♀ (MA = 39.1 ± 10.0 y)    Age and gender matched | - Suspected of having objective tinnitus or somatic TI  - Chronic otitis media, retrocochlear lesions, endolymphatic hydrops, or congenital ear malfunction  - TI accompanied by dizziness  - History of ototoxic drug use | ABR  - amp of wave I and V  - wave V/I amp ratio  100 µs click stimuli, 90 dB nHL, 13 clicks/s, bandpass filtered 100-3000 Hz, 1500 sweeps  Average data from two trials | Analyzed separately according to sex. In both ♂ and ♀: no sign diff in amp of wave I or wave V or wave V/I amp ratio |  |
| Song et al., Otology & Neurology, 2018 (95) | TI (n = 20)  - 10 ♂, 10 ♀  - MA = 37 ± 12.6 y  - CNH  - 8 UL TI (3 R, 5 L), 12 BL TI  - THI, laterality and onset of TI collected | No TI, CNH (n = 91)  - 40 ♂, 51 ♀  - MA = 43 ± 11.3 y  - CNH | - Hearing threshold > 20 dB at least once for frequencies of 0.25 to 8 kHz  - incomplete medical records | ABR (lat and amp waves I, III, and V; IPL I-III, III-V, and I-V; amp ratio III/I and V/I)  90 dB click stimulus | - lat of wave V on L side sign ↓ in TI  - amp wave I on L sign ↓ in TI | Retrospective  Left and right side analyzed seperately |
| ABR-MLR | | | | | | |
| Bilgen et al., Journal of International Advanced Otology, 2010 (56) | UL TI (n = 22)  - 2 ♂, 20 ♀  - MA = 34.4 y  - CNH  - UL TMD (grade 2 in all patients according to Helkimo’s Clinical Dysfunction Index) | No TI (n = 15)  - 5 ♂, 10 ♀  - MA = 29.4 y  - CNH  - No info on matching | - BL TMD  - Abnormal otoscopic findings and audiograms  - Otological disorder | - ABR (lat and amp of waves I, III, and V)  - MLR (lat and amp of waves Na, Pa, Nb, Pb, and Nc)  Click sounds, 9.7 clicks/s, 90 dB nHL. 4 averages of 1000 stimuli per ear were calculated.  ABR bandpass filter: 30-3000 Hz  MLR bandpass filter: 10-250 Hz | - ABR: BL no diff in lat and amp of waves I, III, V  - MLR ipsilateral ears: BL sign shorter latencies of wave Pb, Nc and greater waves Na, Pa and Pb in UL TI & UL TMD | Population of TI with TMD |
| MLR | | | | | | |
| Dos Santos-Filha et al., Medical Science Monitor, 2015 (16) | TI (n = 30)  - 26 ♂, 4 ♀  - MA = 41 y  - CNH  - Exposed to occupational noise (> 85 dBA) | No TI (n = 30)  - 26 ♂, 4 ♀  - MA = 41.6 y  - CNH  - Exposed to occupational noise (> 85 dBA)  No info on matching | - Neurological, psychiatric, and/or behavioural disorders | - MLR: lat and amp of waves Na, Pa; Na-Pa amplitude  Monaural click stimuli, 70 dBHL, 9.9 clicks/s, 1,000 stimuli  Bandpass filter: 10-300 Hz | TI vs control:  - No sign diff between TI and no TI in terms of normal and abnormal Na and Pa wave amp and lat  - both groups ↑ lat for Na and Pa waves |  |
| Theodoroff et al., International Tinnitus Journal, 2011 (57) | Severe TI (n = 14)  - 10 ♂, 4 ♀  - MA = 50.3 ± 12.7 y  - CNH  - THI score: range 44-98  - Mean TI duration: 9.7 ± 9.2 y | No TI (n = 14)  - 6 ♂, 8 ♀  - MA = 40.5 ± 13.2 y  - CNH  No info on matching | - History of neurological disease  - Significant hearing loss from 0.25 to 3 kHz  - Middle ear pathology | MLR  - Absolute lat Na, Pa, Nb, Pb  - Relative amp Na-Pa and Nb-Pb  Click stimulus, 100 µs, rarefraction polarity, rate 1.1/sec, 70 dB nHL.  Evoked responses amplified with a gain of 75 K and band-pass filtered 0.3-1.5 kHz over a 106.6 ms time window. Averaged in trials of 500 sweeps | No sign diff |  |
| ABR-FFR | | | | | | |
| Guest et al., Hearing Research, 2017 (58) | TI (n=20)  - 10 ♂, 10 ♀  - MA = 25.7 ± 1.3 y  - CNH  - > 4 months stable TI, non-pulsatile  - TFI = 33 ± 7 | No TI (n=20)  - MA = 25.5 ± 1.3 y  - CNH  Matched for age and sex | - Abnormal PTA thresholds (> 20 dB HL)  - Abnormal middle ear function  - History of head trauma, middle ear surgery, neurological disorder, or ototoxic exposure | ABR (wave I and V amp, wave I/V amp ratio)  FFR (EFR) (subcortical)  ABR: Rate Click stimuli with a 10 dB bandwidth extending from about 1.2 to 4.7 kHz  alternated clicks between ears, 7.05 clicks/s in each ear, with an overall presentation rate of 14.1/s, total 7,040 presentations per ear.  EFR: variable-modulation-depth paradigm. Stimuli: 75 dB SPL transposed tones, with a 4000 Hz carrier and 100 Hz modulator. Stimulus duration: 400 ms + 15 ms onset and offset ramps. Four stimuli in sequence: 0 dB, inverted 0 dB, -6 dB, inverted –6 dB. Average interstimulus interval 400 ms, jittered by up to 10%. Sequence presented 630 times. | - ABR: No sign diff in wave I, wave V amp and wave I/V ratio  - FFR (EFR): TI subjects producing lower response amplitudes than controls, but non-sign  - No correlations between ABR/EFR and noise exposure |  |
| FFR | | | | | | |
| Paul et al., Hearing Research, 2017 (59) | TI (n = 13)  - 4 ♂, 9 ♀  - MA = 23.2 ± 6.15 y  - CNH  - Chronic BL TI  - Mean TI duration: 10.7 ± 9.9 y  - Mean THQ score: 17.6 ± 9.9  - TL: 8.2 dB SL | No TI (n = 24)  - 5 ♂, 20 ♀  - MA = 19.5 ± 2.82 y  - CNH  No info on matching | - History of head trauma  - Use of medication | FFR (EFR)  EFR power recorded as dB SNR at 5 stimulus conditions: AM tone (5kHz tone at 75 dB SPL) in quiet, AM tone within NBN (to diminish contribution of high-SR fibers), in subsequent conditions AM depth was further reduced to –2.5 dB and –6dB, NBN alone (no AM) | TI patients had smaller EFRs than controls. Post-hoc contrasts of the main effect were not significant for any individual stimulus condition, although the 0 dB condition and the –2.5 dB AM, NBN condition appeared to have contributed most to the overall group effect  --> AM encoding was poorer overall in TI, suggesting greater synaptic loss in TI group |  |

## Mixed population

| **Reference (first author, journal citation, year)** | **Subjects** | **Control** | **Exclusion criteria** | **Outcome measure** | **Results** | **Comments** |
| --- | --- | --- | --- | --- | --- | --- |
| ABR | | | | | | |
| Gilles et al., Frontiers in Neuroscience, 2016 (52) | TI, noise-induced (n=19)  - 11 ♂, 8 ♀  - University students  - Attend parties, concerts or festivals on a weekly basis  - Mean TQ: 27.72 ± 15.23  - Mean VAS loudness: 5.44 ± 2.46 | No TI (n=68)  - 12 ♂, 56 ♀  - Attend parties, concerts or festivals on a weekly basis  Matching by age, gender, and pure-tone thresholds  ABR performed in 23 controls | - Pulsatile tinnitus  - Middle ear pathology  - Known neurologic diseases  - History of depression  - Asymmetric sensorineural hearing loss  - ≥ 30 years old | ABR (amp and lat waves I, III, V; IPL I-III, I-V, III-V; interpeak amp ratios I-III, I-V and III-V)  100 µs-duration click stimuli, alternating polarity, 31.0 clicks/s, 80 dBnHL. Contralateral white noise masking 55 dBnHL.  Band pass filter: 100-3000 Hz.  Artefact rejections 23.8 µV, maximum of 2000 averages | No stat differences between TI subjects and controls with frequent recreational noise exposure | Aim was to reveal early signs of recreational noise damage in young adults |
| Gu et al., JARO, 2012 (86) | TI (n=15)  - All ♂  - MA = 42 ± 6 y  - Different types of TI  - TI pitch, loudness, MML and presence of residual inhibition assessed  - Audiograms generally within normal limits | 1) No TI (n = 21)  - All ♂  - MA = 43 ± 7 y  - Audiograms generally within normal limits  Matched for age and hearing sensitivity    2) No TI but younger (n = 11)  - All ♂  - MA = 23 y ± 2  - Audiograms generally within normal limits  No info on matching | - ♀  - Variation in sound level tolerance  - Depression  - Anxiety | ABR (lat and amp of waves I, III, V; amplitude ratios III/I and V/I)  100 µs click stimuli, monaurally at 30, 50, 70, and 80 dB nHL. Opposite ear: boradband noise at 10, 30, and 40 dB nHL, respectively for 50, 70, and 80 dB nHL.  Rate 11 clicks/s, 4-min runs, interval 10% (9 ms). Six runs at 30 dB, 3 runs at each of the higher stimulus levels, yielding 15,840 and 7,920 total click presentations per stimulus level respectively. | - TI vs age matched control:  ↓ wave I amp  ↑ waves III and V amp  ↑ III/I and V/I amp ratios    - TI and matched non-TI group vs younger control:  Both groups ↑ thresholds above 4 kHz and ↓ wave I amplitude  → differences between TI and matched non-TI subjects occurred against a backdrop of shared peripheral dysfunction  - No correlations between SLTQ score or LDL and ABR amp or amp ratios  - Correlations between wave I amp and amp ratios, and depression and anxiety at different dB |  |
| Ikner et al., Ear and Hearing,1990 (62) | TI (n = 35)  - 19 ♂, 16 ♀  - MA = 40 y | No TI (n = 35)  - 18 ♂, 17 ♀  - MA = 36 y  Matched for age, gender, and ear function (auditory reflex) | - Abnormal middle ear function  - PTA > 25 dB  - Unidentifiable wave forms at 75 dB stimulus level | ABR (lat waves I, III, and V; IPL I-III, III-V, and I-V)  100 µs click stimuli, monaurally, 75 dBnHL, 21.9 clicks/s | In acoustic reflex threshold (ART) match  - sign ↑ lat for waves I, III and V in TI  - IPL III-V and I-V also elevated (p<0.02) in TI  By gender  - In TI ♀: in 1 to 4 kHz matched and normal-hearing subjects: ↑ lat wave I |  |

## Hearing loss

| **Reference (first author, journal citation, year)** | **Subjects** | **Control** | **Exclusion criteria** | **Outcome measure** | **Results** | **Comments** |
| --- | --- | --- | --- | --- | --- | --- |
| ABR | | | | | | |
| Attias et al., Hearing Research, 1993 (96) | TI (n=12)  - all ♂  - 26-45 years  - chronic TI > 5 years  - career army personnel with history of repeated noise exposure  - NIHL  - BL TI, TP 5-8 kHz and TL 10-20 dB SL | No TI (n=12)  - all ♂  - military personnel  - NIHL  Age, and hearing loss matched | - Major psychiatric disorder or depressive symptomatology  - Pharmacological treatment  - Alcohol or drug abuse | ABR (lat and amp of waves I, III, and V; IPL I-III, III-V, and I-V)  1024 alternating polarity clicks of100 µs, 120 dB SPL, rate 10 clicks/s, presented bilaterally, bandpass filter 200-3000 Hz  + EEG recordings for CAEPs | - No differences  - All within normal limits | Research focuses on CAEPs |
| Attias et al., Audiology, 1996 (97) | TI (n=13) (21 ears)  - all ♂  - MA = 35 y  - chronic TI > 5 years  - 8 BL, 5 UL = 21 ears  - Noise induced TI  - Active career military personnel with history of repeated noise exposure  - NIHL  - TP: n=4: 3-4 kHz; n = 17: 6-12 kHz  - TL: n=8: 0-3 dB SL; n =7: 4-5 dB SL, n=6: 7-10 dB SL | No TI (n=11) (21 ears)  - Active career military personnel with history of repeated noise exposure  - NIHL  Age and hearing matched | - Medication or drug abuse  - Neurological condition | ABR (lat and amp of I, III, and V; amp ratio III/I and V/III, measured ipisilateral and contralateral to stimulated ear)  Alternating polarity click stimuli of 100 µs, rate of 10.3/sec, 120 dB SPL presented monaurally  Filter: 100-2000 Hz | - Ipsilaterally recorded wave III amp significantly larger in TI patients vs controls  - Ipsilateral III-I amp ratio sign larger for TI group  - Other waves did not differ |  |
| Pinkl et al., International Tinnitus Journal, 2017 (55) | TI (n = 11) 21 ears  1) TI normal hearing (n = 10 ears)  2) TI hearing loss within 2000-4000Hz (n = 11 ears)  - ♂/♀ not given  -MA 46.5 ± 19.15 y  -THI 22.8 | No TI (n = 10)  - Monaurally tested = 10 ears  - ♂/♀ not given  - MA: 24.4 ± 2.0 y | - Middle ear pathology  - Ototoxic medications  - Ear surgery | ABR (lat of waves I, III, V; amp wave V; IPL III-I, V-III, and V-I)  Click stimuli and tone burst stimuli (tone burst frequency chosen based on TP frequency), 85 dB nHL, 1,024 sweeps, sample rate of 512 Hz. Bandpass filter: 100-3000 Hz. | - No sign diff in amp and lat between TI with and without hearing loss except for delayed IPL V-I in TI with NHL  - No sign diff in amp and lat between TI with normal hearing and controls except for ↑ IPL V-III in TI with NHL  - Lat wave V ↑ and IPL V-III ↑ in TI with hearing loss compared to controls |  |
| Rosenhall et al., Scandinavian Audiology, 1995 (98) | TI (n = 113)  1) normal or only slightly impaired hearing (n = 56)  - 30 ♀, MA 43.8 ± 14.0 y  - 26 ♂, MA 40.5 ± 13.7 y  2) Hearing loss (45-60 dB at 4 kHz) (n = 57)  - 20 ♀, MA 59.5 ± 12.2 y  - 37 ♂, MA 54.9 ± 9.1 y | No TI (n = 220)  1) normal or only slightly impaired hearing (n = 54)  - 30 ♀  - 24 ♂  2) Hearing loss (45-60 dB at 4 kHz) (n = 166)  - 71 ♀  - 95 ♂  NB: some of them had slight TI  One ear per subject as included in the study | - Retrocochlear lesions | ABR  - lat of wave I, III, and V  - IPLs between waves I-III, III-V and I-V  Rarefration halfsine waves, 80 dBnHL, 20 stimuli/s, 1000 stimuli epochs, filter 150-2000 Hz | Normal or slightly impaired hearing loss  - ♀: lat waves I and V and III-V IPL sign ↑ in TI  - ♂: lat of waves I, III and V sign ↑ in TI  Hearing loss  - ♀: no sign diff  - ♂: lat of waves III and V and III-V IPL sign ↑ in TI | ♀ and ♂ analyzed separately |

**Abbreviations**

AM = amplitude modulation

Amp = amplitude

BL = bilateral

CAEP = cortical auditory evoked potential

CNH = clinically normal hearing

dB HL = decibels hearing level

dB nHL = decibels normal hearing level

dB SL = decibels sensation level

dB SPL = decibels sound pressure level

F1 = First formant frequency range

F0 = fundamental frequency

HF = higher frequency region

IPL = interpeak latency

Lat = latency

LDL = loudness discomfort level

MA = mean age

MML = minimal masking level

NBN = narrowband background noise

NIHL = noise induced hearing loss

NTE = non-tinnitus ear

SLTQ = questionnaire assessing sound-level tolerance

THI = tinnitus handicap inventory

TE = tinnitus ear

TI = tinnitus

TL = tinnitus loudness

TP = tinnitus pitch

THI = Tinnitus Handicap Inventory

THQ = Tinnitus Handicap Questionnaire

# S3: Items of risk of bias assessment

To assess the methodological quality of cross-sectional studies, the Joanna Briggs Checklist for Analytical Cross-Sectional Studies (49), which consists of eight items, was used. The following 8 items were assessed as “yes”, “no”, “unclear” or “not applicable”.

**Inclusion criteria**

Were the criteria for inclusion in the sample clearly defined and were they developed prior to recruitment of the study participants?

**Study subjects and settings**

Were the study subjects and the setting described in detail? At least the following characteristics should be described: gender ratio, age (age range and/or mean age and standard deviation of mean age), hearing thresholds, population from which the study participants were recruited (including location and time period). Were more details on tinnitus described (duration of symptoms, severity scored by questionnaires, etc.)?

**Exposure measurement**

Was AEP measurement performed a valid and reliable way? (e.g. valid hardware and software, electrode setup, etc.)? Was AEP acquisition performed by an experienced researcher/clinician?

**Measurement of the condition**

Was the presence of tinnitus (and its duration and characteristics) assessed by a professional (low risk of bias), or did subjects self-report whether they belonged to the tinnitus group (higher risk of bias)?

**Identification of confounding factors**

Were confounding factors identified?

The following possible confounding factors were taken into account within this item: age, gender, hearing loss, use of certain medications (e.g. ototoxic medication, long-time use of anti-epileptic drugs or antidepressants, etc.), middle ear pathology, neurological disorders, psychiatric disorders.

**Dealing with confounding factors**

Were strategies to deal with confounding factors stated?

Were certain confounding factors incorporated in the exclusion criteria?

Were tinnitus patients matched to control subjects? If so, by which patient characteristics was matching performed (e.g. by age, gender, and/or hearing loss)?

**Outcome measurement**

Was AEP analysis performed in a valid and reliable way? Were AEPs processed and interpreted by an experienced researcher/clinician?

**Statistical analysis**

Was appropriate statistical analysis used?

# S4: Results for ABR component

| **Reference** | | **Subjects (n)** | **Controls (n)** | **ABR latencies** | | | | | | **ABR amplitudes** | | | | | |
| --- | --- | --- | --- | --- | --- | --- | --- | --- | --- | --- | --- | --- | --- | --- | --- |
|  |  |  |  | **I** | **III** | **V** | **IPL I-III** | **IPL III-V** | **IPL I-V** | **I** | **III** | **V** | **Ratio III/I** | **Ratio V/III** | **Ratio V/I** |
| **Normal hearing (PTA ≤ 20 dB HL)** | | | | | | | | | | | | | | | |
| **Barnea et al., 1990 (82)** | | 17 | 17 |  |  |  |  |  |  |  |  |  |  |  |  |
| **Bilgen et al., 2010 (56)** | | 22 (+ TMD) | 15 |  |  |  |  |  |  |  |  |  |  |  |  |
| **Cartocci et al., 2012 (89)** | | 10 | 14 |  |  | > * |  | > * |  |  |  |  |  |  |  |
| **Dadoo et al., 2019 (84)** | | 80 ears | 80 ears | > * |  |  |  |  |  |  |  |  |  |  |  |
| **Dos Santos-Filha et al., 2014 (90)** | | 30 | 30 |  |  |  |  |  |  |  |  |  |  |  |  |
| **Gabr et al., 2020 (91)** | | 30 | 30 | > * | > * | > * |  |  |  | ↓ * | ↓ * |  |  |  |  |
| **Guest et al., 2017 (58)** | | 20 | 20 |  |  |  |  |  |  |  |  |  |  |  |  |
| **Hsu et al., 2013 (92)** | | 15 | 15 |  | > * | > * |  |  |  |  |  |  |  |  |  |
| **Kehrle et al., 2008 (67)** | | 13 UL TI , 24 BL TI  = 61 TI ears | No TI (n = 38)  = 76 ears | > * | > * | > * |  | > * |  |  |  |  |  |  | ↑ * |
| **Konadath et al., 2016 (85)** | | 20 | 20 |  |  |  |  |  |  |  |  |  |  |  |  |
| **Makar et al., 2017 (68)** | | 30 | 30 | > * | > * | > * |  | > * |  |  |  |  |  |  |  |
| **Nemati et al., 2014 (83)** | | 25 | 16 |  |  |  |  |  |  |  |  |  |  |  | ↑ * |
| **Omidvar et al., 2018 (60)** | | 18 | 20 |  | > * | > * |  |  | > * |  |  |  |  |  |  |
| **Pinkl et al., 2017 (55)** | | 5 | 10 |  |  |  |  | > * |  |  |  |  |  |  |  |
| **Schaette et al., 2011 (73)** | | 15 | 18 |  |  |  |  |  |  | ↓ * |  |  |  |  |  |
| **Shim et al., 2017 (93)** | | 19 ♂ | 8 ♂ |  |  |  |  |  |  |  |  | ↓ * |  |  |  |
|  |  | 24 ♀ | 10 ♀ |  |  |  |  |  |  |  |  |  |  |  |  |
| **Shim et al., 2021 (94)** | | 12 ♂ | 12 ♂ |  |  |  |  |  |  |  |  |  |  |  |  |
|  |  | 15 ♀ | 15 ♀ |  |  |  |  |  |  |  |  |  |  |  |  |
| **Song et al., 2018 (95)** | | Right ear (n = 20) | Right ear (n = 91) |  |  |  |  |  |  |  |  |  |  |  |  |
|  |  | Left ear (n = 20) | Left ear (n = 91) |  |  | < * |  |  |  | ↓ * |  |  |  |  |  |
| **Mixed population** | | | | | | | | | | | | | | | |
| **Gilles et al., 2016 (52)** | | 19 | 23 |  |  |  |  |  |  |  |  |  |  |  |  |
| **Gu et al., 2012 (86)** | | 15 | 21 |  |  |  |  |  |  | ↓ * |  | ↑ * | ↑ * |  | ↑ * |
| **Ikner et al., 1990 (62)** | | 35 | 35 | > * | > * | > * |  | > * | > * |  |  |  |  |  |  |
| **Hearing loss observed** | | | | | | | | | | | | | | | |
| **Attias et al., 1993 (96)** | | 12 | 12 |  |  |  |  |  |  |  |  |  |  |  |  |
| **Attias et al., 1996 (97)** | | 13 (21 TI ears) | 11 (21 ears) |  |  |  |  |  |  |  | ↑ * |  | ↑ * |  |  |
| **Pinkl et al., 2017 (55)** | | 6 | 10 |  |  | > * |  | > * |  |  |  |  |  |  |  |
| **Rosenhall et al., 1995 (98)** | | Normal/slightly impaired hearing (n = 26 ♂) | 24 ♂ | > * | > * | > * |  |  |  |  |  |  |  |  |  |
|  |  | Normal/slightly impaired hearing (n = 30 ♀) | 30 ♀ | > * |  | > * |  | > * |  |  |  |  |  |  |  |
|  |  | Hearing loss (n = 37 ♂) | 95 ♂ |  | > * | > * |  | > * |  |  |  |  |  |  |  |
|  |  | Hearing loss (n = 20 ♀) | 71 ♀ |  |  |  |  |  |  |  |  |  |  |  |  |
|  | Non-significant longer latency or larger amplitude in tinnitus patients | | | | | | | | | | | | | | |
| > * | Significant (p < 0.05) longer latency in tinnitus patients | | | | | | | | | | | | | | |
| ↑ * | Significant (p < 0.05) larger amplitude in tinnitus patients | | | | | | | | | | | | | | |
|  | No difference in latency or amplitude | | | | | | | | | | | | | | |
|  | Non-significant shorter latency or smaller amplitude in tinnitus patients | | | | | | | | | | | | | | |
| < * | Significant (p < 0.05) shorter latency in tinnitus patients | | | | | | | | | | | | | | |
| ↓ * | Significant (p < 0.05) smaller amplitude in tinnitus patients | | | | | | | | | | | | | | |
|  | This ABR component was not acquired in this study | | | | | | | | | | | | | | |
| IPL = interpeak latency  TI = tinnitus  TMD = temporomandibular dysfunction | | | | | | | | | | | | | | | |

# S5: Reasons for exclusion of papers for meta-analysis

| Reference (first author, year) | AEP components | Reason for exclusion |
| --- | --- | --- |
| Attias et al., 1993 (96) | ABR: latency and amplitude of waves I, III, and V; IPL I-III, III-V, and I-V | Tinnitus patients with hearing loss |
| Attias et al., 1996 (97) | ABR: latency and amplitude of waves I, III, and V; amplitude ratio III/I and V/III | Tinnitus patients with hearing loss |
| Barnea et al., 1990 (82) | ABR: latency and amplitude of waves I, III, V and IPL V-I | Outcome of control group not reported in paper |
| Bilgen et al., 2010 (56) | - ABR: latency and amplitude of waves I, III, and V  - MLR: latency and amplitude of waves Na, Pa, Nb, Pb, and Nc | Tinnitus patients also had TMD, which might also cause electrophysiological changes |
| Dos Santos Filha et al., 2015 (16) | MLR: latency and amplitude of waves Na, Pa; Na-Pa amplitude | Amplitudes and latencies not reported in paper |
| Gilles et al., 2016 (52) | ABR: latency and amplitude of waves I, III, and V; IPL I-III, I-V, and III-V; interpeak amplitude ratios I-III, I-V, and III-V | Presence of clinically normal hearing in tinnitus patients not specifically mentioned in paper |
| Gu et al., 2012 (86) | ABR: latency and amplitude of waves I, III, and V; amplitude ratios III/I and V/I | Tinnitus patients with hearing loss |
| Guest et al., 2017 (58) | - ABR: amplitude of waves I and V  - FFR (envelope following response) | Amplitudes and latencies of ABRs not reported in paper, not enough FFR studies with similar methodology and outcome to compare results with |
| Ikner et al., 1990 (62) | ABR: latency waves I, III, and V; IPL I-III, III-V, and I-V | Tinnitus patients could have hearing loss |
| Nemati et al., 2014 (83) | ABR: latency and amplitude of waves I, III, and V; IPL I-III, III-V, and I-V; amplitude ratio III/I and V/I | Standard deviations of mean ABR latencies and amplitudes not reported |
| Paul et al., 2017 (59) | FFR (envelope following response) | Not enough studies with similar methodology and outcome to compare results with |
| Rosenhall et al., 1995 (98) | ABR: latency of wave I, III, and V; IPL I-III, III-V, and I-V | Specific latencies and IPLs not reported in paper |
| Shim et al., 2017 (93) | ABR: latency and amplitude of wave I and V, IPL I-V, amplitude ratio wave V/I | Specific amplitudes and latencies not reported in paper |
| Shim et al., 2021 (94) | ABR: amplitude of wave I and V, amplitude ratio V/I | Specific amplitudes and latencies not reported in paper |
| Song et al., 2018 (95) | ABR: latency and amplitude of waves I, III, and V; IPL I-III, III-V, and I-V; amplitude ratio III/I and V/I | Separate results for left and right ear |
| Theodoroff et al., 2011 (57) | MLR: latency and amplitude of wave Na, Pa ; Na-Pa amplitude | Amplitudes and latencies not reported in paper |
| ABR = auditory brainstem response  FFR = frequency-following response  IPL = interpeak latency  MLR = middle-latency response  TMD = temporomandibular dysfunction | | |

# S6: Study population characteristics of studies included in meta-analysis

| **Reference (first author, year)** | **Number of participants (n)** | | **Mean age (range)** | | **Proportion of men** | | **Mean tinnitus duration (months)** | **Mean THI score** |
| --- | --- | --- | --- | --- | --- | --- | --- | --- |
|  | **TI** | **Con** | **Tinnitus** | **Controls** | **Tinnitus** | **Controls** |  |  |
| **Cartocci et al., 2012 (89)** | 10 | 14 | 43.9 | 45.1 | 1.0 | 1.0 |  |  |
| **Dadoo et al., 2019 (84)** | 40 | 40 | 33.4 | 30.6 | 0.58 | 0.58 | 6.47 |  |
| **Dos Santos Filha et al., 2014 (90)** | 30 | 30 | 41.0 (27-50) | 41.6 (27-50) | 0.87 | 0.87 |  |  |
| **Gabr et al., 2019 (91)** | 30 | 30 | (18-40) | (18-40) |  |  | 69.6 | 35.2 |
| **Hsu et al., 2013 (92)** | 15 | 15 | 41.1 | 37.9 | 0.47 | 0.33 |  |  |
| **Kehrle et al., 2008 (67)** | 37 | 38 | 36.0 |  |  |  |  |  |
| **Konadath et al., 2016 (85)** | 20 | 20 | 33.2 (20-48) | 20.5 (18-22) | 0.50 | 0.50 |  | > 38 |
| **Makar et al., 2017 (68)** | 30 | 30 | 39.6 | 37.6 | 1.0 | 1.0 | 15.06 |  |
| **Omidvar et al., 2018 (60)** | 18 | 20 | 38.1 | 35.6 | 0.50 | 0.45 |  | n = 9: THI < 16  n = 9: THI 18-36 |
| **Pinkl et al., 2017 (55)** | 5 | 10 | 46.5 | 24.4 |  |  |  | 22.8 |
| **Schaette et al., 2011 (73)** | 15 | 18 | 36.3 | 33.2 | 0.00 | 0.00 |  |  |
| **Mean over all studies** | 27 | 28 | 38.9 | 34.1 | 0.61 | 0.59 | 30.4 |  |

# S7: Investigation of influential studies

Influential studies or outliers for each ABR component were identified based on Cook’s distances. An overview of the identified influential studies is provided in the table below (S7 table).

**S7 table: Influential studies identified for each ABR component.**

| **ABR component** | **Influential studies** | **Results after removal of influential studies** |
| --- | --- | --- |
| **Latency wave I** | None | N/A |
| **Latency wave III** | Kehrle et al., 2008 (62) | No differences compared to the primary analysis |
| **Latency wave V** | Kehrle et al., 2008 (62) | No differences compared to the primary analysis |
| **Interpeak latency (IPL) I-III** | None | N/A |
| **Interpeak latency (IPL) III-V** | None | N/A |
| **Interpeak latency (IPL) I-V** | None | N/A |
| **Amplitude wave V** | None | N/A |

As for latency of wave I, visual inspection showed that the study of Pinkl et al. (55) might be a potential outlier. Thus, this study was also removed for post hoc analysis.

For all components, the removal of influential papers and outliers did not result in a different outcome for these post hoc analyses compared to the primary analyses. Forest plots with the influential papers and outliers excluded are given in S7 figure.


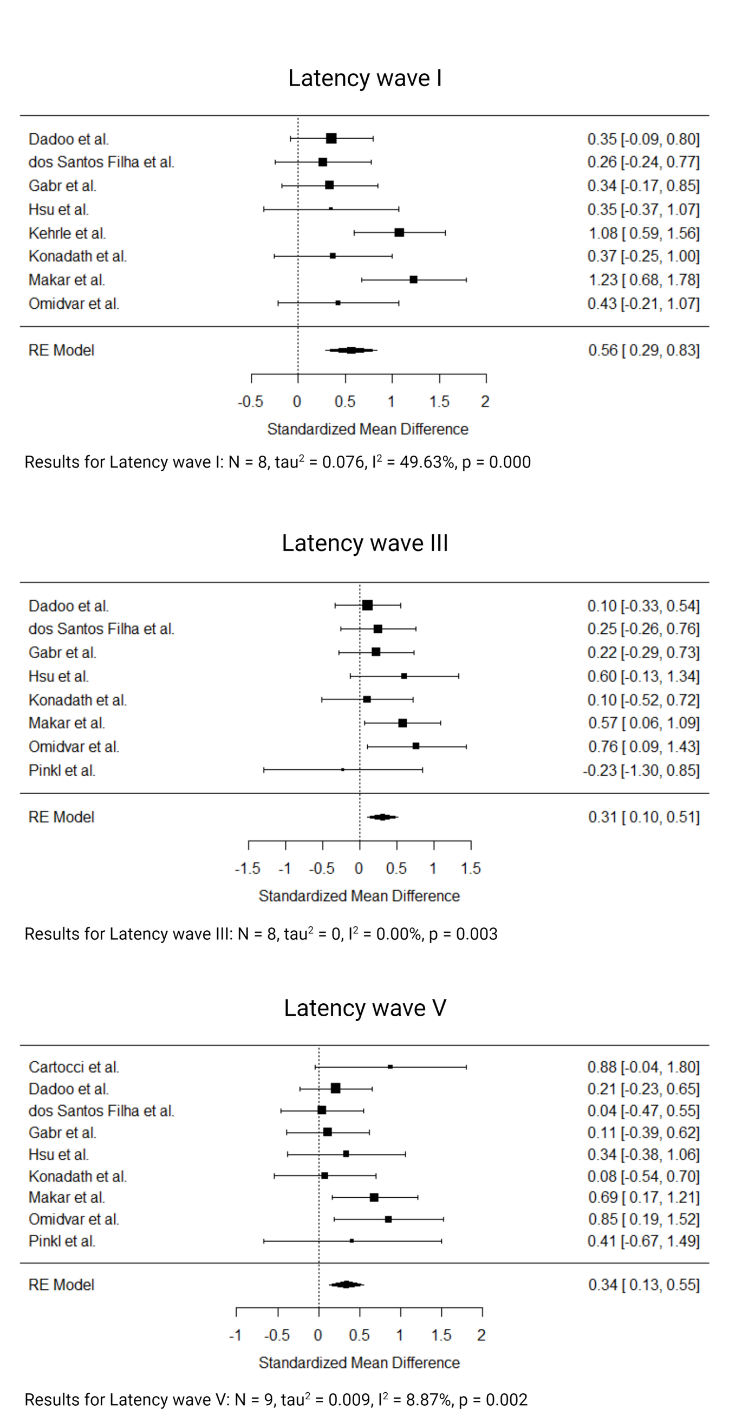


**S7 Figure.** Forest plots for latencies of waves I, III, and V with the influential studies and outliers excluded. Results did not differ from the primary analysis.

# S8: Investigation for publication bias


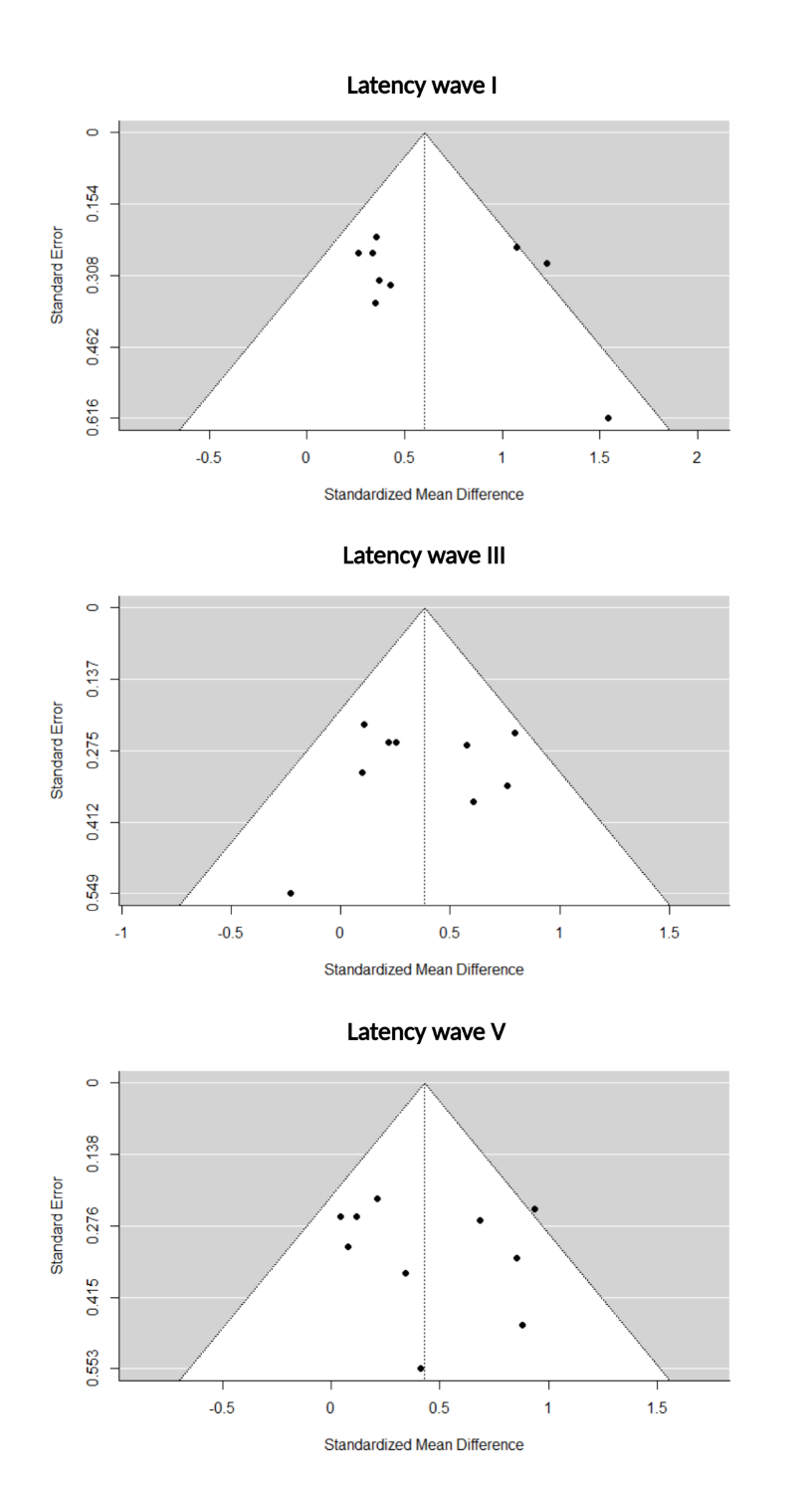


**S8 figure.** Funnel plots for ABR latencies of waves I, III, and V and amplitude of wave I.

# S9: Results for MLR component

| **Reference** | **Subjects (n)** | **Control (n)** | **Latencies** | **Amplitudes** |
| --- | --- | --- | --- | --- |
| **Normal hearing (≤ 20 dB HL)** | | | | |
| **Bilgen et al., 2010 (56)** | 22 (+ TMD) | 15 | Na: No significant difference  Pa: No significant difference  Nb: No significant difference  Pb: Significantly shorter  Nc: Significantly shorter | Na: Significantly larger  Pa: Significantly larger  Nb: No significant difference  Pb: Significantly larger  Nc: No significant difference |
| **Dos Santos-Filha et al., 2015 (16)** | 30 | 30 | Na: No significant difference  Pa: No significant difference | Na-Pa: No significant difference |
| **Theodoroff et al., 2011 (57)** | 14 | 14 | Na: No significant difference  Pa: No significant difference  Nb: No significant difference  Pb: No significant difference | Na-Pa: No significant difference  Nb-Pb: No significant difference |
